# Exploring Wettability of Liquid Iron on Refractory Oxides with the Sessile Drop Technique and Density Functional-Derived Hamaker Constants

**DOI:** 10.1021/acsami.4c21877

**Published:** 2025-02-28

**Authors:** Sudhanshu Kuthe, Mathias Boström, Wen Chen, Björn Glaser, Clas Persson

**Affiliations:** † Department of Materials Science and Engineering, KTH Royal Institute of Technology, SE-100 44 Stockholm, Sweden; ‡ Centre of Excellence ENSEMBLE3 Sp. z o. o., Wolczynska Str. 133, 01-919 Warsaw, Poland; § UniversalLab GmbH, Park Innovaare: deliveryLAB, 5234 Villigen, Switzerland; ∥ Chemical and Biological Systems Simulation Lab, Centre of New Technologies, University of Warsaw, Banacha 2C, 02-097 Warsaw, Poland; ⊥ Yangtze Delta Region Institute of Tsinghua University, Zhejiang 314006, China

**Keywords:** wettability, Hamaker constant, contact angle, liquid iron, refractory oxide, Casimir–Lifshitz energy, dielectric function, sessile drop method

## Abstract

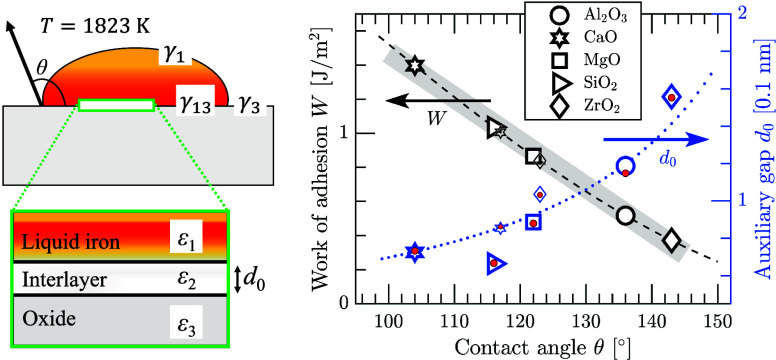

Macroscopic interactions of liquid iron and solid oxides, such as alumina, calcia, magnesia, silica, and zirconia, manifest the behavior and efficiency of high-temperature metallurgical processes. The oxides serve dual roles, both as components of refractory materials in submerged entry nozzles and also as significant constituents of nonmetallic inclusions in the melt. It is therefore crucial to understand the physicochemical interplay between the liquid and the oxides in order to address the nozzle clogging challenges and thereby optimize cast iron and steel production. This paper presents a methodology for describing these interactions by combining the materials’ dielectric responses, computed within the density functional theory, with the Casimir–Lifshitz dispersion forces to generate Hamaker constants. The approach provides a comprehensive understanding of the wettability of iron against these refractory oxides, revealing the complex relation between the molecular and macroscopic properties. Our theoretically determined crystalline structures are confirmed by room-temperature X-ray diffraction, and the contact angles of liquid iron on the oxides are validated with a sessile drop system at a temperature of 1823 K. For comparison, we also present the wettability of the oxides by a liquid tin–bismuth alloy. The findings are essential in advancing the fundamental understanding of interfacial interactions in metallurgical science and pivotal in driving the development of more efficient and reliable steelmaking processes.

## Introduction

Iron and steel are essential materials in various sectors due to their versatile properties, ranging from construction to automotive and aerospace industries. The production of high-quality steel is a complex process, and one of the main challenges faced during production is the clogging of submerged entry nozzles (SEN) due to the interaction between liquid iron (Fe(liq)) and various refractory oxides, including alumina (Al_2_O_3_), calcia (CaO), magnesia (MgO), silica (SiO_2_), and zirconia (ZrO_2_). The clogging phenomena are significantly influenced by the low wettability of these refractory oxides with molten steel, making it a critical factor in controlling continuous casting processes. The wettability is governed mainly by the interfacial interaction between molten steel and the refractory materials, which is influenced by their individual material properties and operating temperature. Previous studies have focused on experimental characterization and phenomenological models, which, while important, do not provide a full understanding of the molecular mechanisms governing these interactions.

In this work, we present a first-principles approach to study the wettability of liquid Fe on various refractory oxides at the steelmaking temperature *T* = 1823 K (∼1550 °C). We combine density functional theory (DFT) to compute dielectric responses with a Casimir–Lifshitz model to calculate the dispersion forces in order to determine the interactions across the interfaces of material systems. Motivated by the challenges faced in mitigating SEN clogging, we seek to extend the understanding of the interactions by utilizing atomistic calculations together with modeling the macroscopic electromagnetic modes by dispersion forces. The fundamental principles for modeling weak interactions in materials at different length scales are discussed by for instance Fiedler et al.^1^ Furthermore, we verify the crystalline structures of the oxides by X-ray diffraction and measure the contact angles of the Fe(liq)-on-oxide systems by a sessile drop method. The contact angle, a key property in the description of the wettability of the substrate, is governed by cross-interface forces. In the analyses, we determine the related Hamaker constants^2^ by employing the DFT dielectric response functions in the calculations of Casimir–Lifshitz free energies. This proposed methodology serves as an essential component in understanding the interplay between the liquid and oxide particles or surfaces. It will enable the prediction of wettability at high temperatures as well as provide deep insight into the interactions at a molecular level. This approach is particularly useful for systems where direct measurement of surface and interfacial energies is challenging such as Fe(liq)/oxide interfaces at high temperatures.

By integrating our theoretically determined Hamaker constants with the experimental data of the contact angles, we analyze and compare the wettability of different refractory oxides by liquid Fe. Since it is more convenient to experimentally explore liquid–oxide interaction at a lower temperature, we present also the wettability of a liquid tin–bismuth alloy on the oxides at 473 K (∼200 °C).^3,4^ From the analyses of wettability, we propose useful relations for describing the Hamaker constant and the cross-interface forces in terms of the oxides’ high-frequency dielectric constants, and we show that those relations are applicable to two rather different types of liquid metals. Moreover, our findings suggest that the interaction energies are crucial in describing the wettability, and the Hamaker constants should therefore be accompanied by a consistent and complete description of the liquid–solid system, especially when modeling the interactions at close distances. Combining atomistic DFT with large-scale dispersion forces is an appropriate approach for modeling the weak interactions in these systems.

In-depth understanding of the interfacial interactions is critical for further advances in material processing techniques. Our investigation of wettability aims to support the fundamental understanding of submerged entry nozzle clogging phenomena, and we therefore focus on the interaction of liquid iron with the five main refractory oxides and at the specific temperature of steelmaking.^5−8^ Nonetheless, the proposed methodology can be applied over a wide temperature range and for various macro- and mesoscopic liquid–solid systems where weak interaction plays an important role, for example, capillary action in hydrology, plants and wicking fabrics,^9,10^ fluid lubricants,^11,12^ modulating protein adsorption to prevent surface-induced thrombosis from medical devices that are in contact with blood,^13,14^ oleophobic and hydrophilic coatings for antifogging and oil–water separation,^15,16^ treatment of contaminated water,^17,18^ as well as hydrophobicity for rain-repellent or self-cleaning surfaces, protection from moisture and corrosion in electronics, and impregnation of concrete constructions.^19−21^

## Theoretical Details

**Figure 1:**
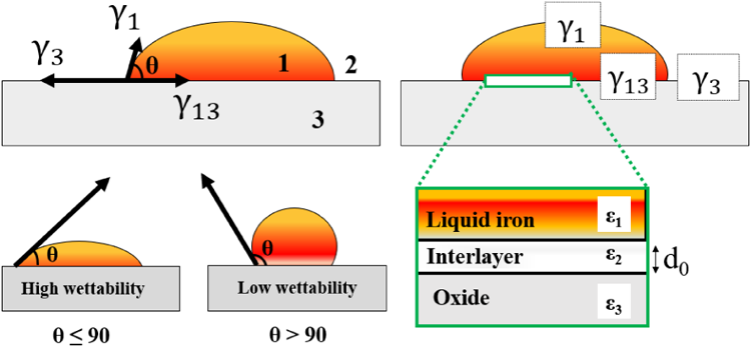
Work of adhesion *W* is determined from the interfacial energy γ_13_, the surface tension γ_1_ of the liquid iron, and the surface energy γ_3_ of the refractory oxide. The material properties are represented by the dielectric function ε_i_(ω). We model the Casimir–Lifshitz cross-interface interactions with a three-layer system, Fe(liq)//oxide, where the interlayer describes an auxiliary gap with thickness *d*_0_ between the liquid iron and the oxide surface. The resulting contact angle, θ is a measure of the wettability.

The applied methodology utilizes the permittivity of the material, describing the response to external electromagnetic field, to determine the cross-interface interaction from the change in zero-point energies associated with the surface modes.^22^ We relax the crystalline structure of the materials and compute their complex dielectric functions with the Kohn–Sham approach within the DFT. From the Kramers–Kronig relation, the dielectric functions are evaluated on the imaginary axis for the temperature-dependent Matsubara frequencies. The response functions thus are the basis for computing the Casimir–Lifshitz free energies and corresponding forces. From this, we determine the DFT-derived Hamaker constants and relate them to the contact angles θ within Young’s theory for ideal surfaces:^23^ γ_1_ cos θ + γ_13_ = γ_3_, where the gravitational forces are neglected and γ is defined by Figure 1. Here, the work of adhesion *W* = γ_1_ + γ_3_ – γ_13_ is a critical parameter, quantifying the energy associated with the adhesion of two different materials. The work of adhesion can be derived from the Hamaker constant *A*_123_ by introducing an auxiliary gap distance *d*_0_ between the two interacting surfaces: *W* = *A*_123_/12π*d*_0_^2^.^24,25^ With this approach, the interfacial description of the Fe(liq)/oxide system becomes a three-layer model Fe(liq)/interlayer/oxide, where the thickness of the intermediate layer is *d*_0_. From our results, we will argue against the notion of a universal value for *d*_0_ for all oxides and instead emphasize that the different characteristics of each system are distinct and important.

### DFT Modeling of Materials

The atomistic calculations are performed within the DFT framework, employing the projector augmented wave method with *GW*-type core potentials. Given the wide-gap insulating character of the oxides, we employ the generalized gradient approximation that has been revised for solids by Perdew et al. (PBEsol).^26^ We correct the PBEsol band gap energy with the hybrid functional by Heyd, Scuseria, and Ernzerhof (HSE)^27^ with a 30% Fock exchange. These computations are carried out utilizing the Vienna Ab initio Simulation Package (VASP).^28,29^ Spin–orbit coupling is neglected because the spin-split of the oxides is small, it affects the band dispersion only locally, and its contribution to the total energy is marginal. Its impact on the effective carrier masses could be strong,^30^ but that will not be reflected on the overall optical properties nor on the dielectric responses.

The valence configurations of the elements should include semicore states and energy transitions associated with high-energy photons. Therefore, we choose Al: 2s^2^p^6^3s^2^p^1^ (see also discussion in ref (31)), Ca: 3s^2^p^6^4s^2^, Fe: 3s^2^p^6^d^6^4s^2^, Mg: 2s^2^p^6^3s^2^, O: 2s^2^p^4^, Si: 2s^2^p^6^3s^2^p^2^, and Zr: 4s^2^p^6^d^2^5s^2^. The compounds are described by their primitive cells, and the irreducible Brillouin zones are typically sampled by 6 × 6 × 6 **k**-meshes. A quasi-Newton variable metric algorithm is utilized for the structural relaxation with a cutoff energy of 800 eV and to an accuracy of 10^–4^ eV/Å for the forces on each atom. Since the optical properties depend on the split-off energies, and the accuracy of determining these energy states depends on turn on bond lengths and bond angles,^32^ we relax the lattice parameters and the atom positions simultaneously. Thereafter, the charge density is generated with a 600 eV cutoff energy, employing Blöchl’s linear tetrahedron integration for the oxides and the Gaussian smearing technique for iron and iterated in the electronic self-consistent loop to reach an energy accuracy of 10^–6^ eV. Solid Fe is paramagnetic just below the melting point, crystallized in a body-centered cubic (bcc) structure.^33^ Therefore, to describe the paramagnetic liquid phase of Fe, we use a simplified model of its atomic configuration. That is, we assume a bcc structure with a weight density corresponding to liquid Fe at 1823 K, namely, 6.978 g/cm^3^. Moreover, the neighboring atoms have opposite spin directions to ensure a perfectly paramagnetic phase. We justify this model by the fact that the surface energy of bcc Fe below the melting point is very close to the surface tension of liquid Fe at 1823 K.^34^ Moreover, one could expect that the ensemble average of atom coordinations and bond lengths should be bcc-like over time, and therefore also the average dielectric response function of liquid Fe should be reasonably close to that of solid Fe(bcc) at *T* ≈ 1800 K, though somewhat smeared out. We therefore employ a Gaussian smearing of *k*_B_*T* = 0.16 eV, where *k*_B_ is Boltzmann’s constant, which is relevant for the considered temperature. Considering some inexactness in measurements at high temperatures, we show that the resulting response function actually agrees rather accurately with the experimental data.

From the electronic structures of the compounds, the imaginary part of the macroscopic dielectric function ε(ω) = ε′(ω) + iε″(ω) is calculated. With the independent single-electron eigenfunctions, the response function has contributions from electronic interband transitions (eq 1), the Drude model of intraband transitions for the metals (eq 2), and ionic vibrations for the oxides (eq 3). Local field effects are neglected. In the long-wavelength limit, the respective parts read
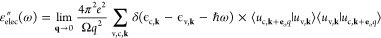
1
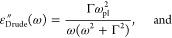
2
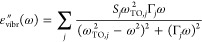
3for the three Cartesian directions **e**_α_. ℏ is the reduced Planck constant, Ω is the unit-cell volume, and *u*_v/c_ is the cell periodic part of the valence (v) or conduction (c) state eigenfunction with the energy ϵ_v/c,**k**_. For the oxides, the accuracy of especially electronic contribution can depend strongly on the size of the **k**-point grid,^35^ and we therefore use a 12 × 12 × 12 **k**-mesh although the values of the low-frequency dielectric constants are sufficiently converged already for the charge density from the 6 × 6 × 6 mesh. The dipole-active longitudinal optical (LO) phonons and the corresponding transverse optical (TO) modes contribute to the dielectric response as vibrations build up an electric field that screens the carriers. Γ_*j*_ is the damping and *S*_*j*_ is the oscillator strength of the *j*th mode in its vibration direction. From the density functional perturbation theory, we compute the Hessian matrix of ionic displacements to model the optical phonons. For metallic Fe, the accuracy in determining the unscreened plasma frequency ω_pl_, which describes the screening from the nearly free carriers in the partially occupied bands, requires a large 60 × 60 × 60 **k**-mesh. The total imaginary part of the dielectric function is the sum of the contributions: the electronic and Drude for metals and the electronic and ionic for the oxides. For the average response function, we take the arithmetic mean of the three Cartesian directions.

Quantities related to interaction energies can be obtained directly from the imaginary part of the dielectric function.^22,25,36^ With the finite temperature formalism in terms of Matsubara frequencies ω_*n*_ = *n*2π*k*_B_*T*/ℏ (with *n* = 0, 1,···), the response function is evaluated on the imaginary axis by the Kramers–Kronig relation (eq 4)^37^
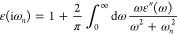
4This spectrum is real-valued and decays smoothly toward one at high frequencies ω_*n*→∞_.

### Casimir–Lifshitz Dispersion Forces

The Casimir–Lifshitz contribution to the interaction free energy between two surfaces across a gap of thickness *d*_0_ (see Figure 1) can formally be calculated by^22^
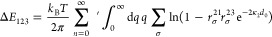
5where σ = transverse electric (TE) and transverse magnetic (TM) in eq 5. The prime in the sum indicates that the first term (*n* = 0) should be weighted by 1/2, and the upper limit of the summation is about 500 in our calculations. The Fresnel reflection coefficients (eq 6) between surfaces *i* and *j* for the transverse magnetic (TM) and transverse electric (TE) polarizations are given by
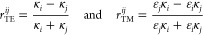
6where κ_*i*_ = (*q*^2^ + ε_*i*_ω_*n*_^2^/*c*^2^)^1/2^ with *i* = 1, 2, and 3, and where *c* is the speed of light. This produces an important contribution to the surface free energies. However, when the two surfaces are very close, only the nonretarded transverse magnetic surface modes contribute.

The work of adhesion is the energy required to separate the two surfaces, which here is directly proportional to the Hamaker constant. In our analysis, we compute the Hamaker constants (eq 7) by means of the Casimir–Lifshitz interactions in the nonretarded limit and with the variable substitution *q* ≡ *z*/*d*_0_
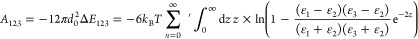
7employing the DFT dielectric response functions of the liquid iron (ε_1_), ideal air (ε_2_ = 1), and the oxides (ε_3_). We denote this three-layer system with the auxiliary interlayer by Fe(liq)//oxide, i.e., with a double-forward slash symbol. There is an alternative approach to describe interface interactions involving only two layers and thus with no auxiliary gap.^38^ However, that model is associated with limiting wave-vectors for the collective surface oscillations, and those parameters are not unproblematic to consider as being dependent on the type of material and type of contribution to the dielectric functions.

The thickness of the intermediate layer (eq 8) is obtained by combining Young’s equation with the work of adhesion, as described by Dupré,^39^ yielding
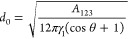
8

## Experimental Details

The primary purpose of room-temperature X-ray diffraction (RT-XRD) in this study was to validate the lattice constants obtained from DFT relaxation of the crystal structures. The RT-XRD analysis was specifically conducted on refractory oxide powder particles. The measurements were performed using a Bruker D2 diffractometer, with a scanning range of 5–90°, a scan speed of 5°/min, and a step size of 0.02°. The RT-XRD source operated at 40 kV and 15 mA, utilizing copper Kα radiation at a wavelength of approximately 0.154 nm.

The contact angles were measured with the support of a high-temperature sessile drop experimental setup (Figure S1). A horizontal Entech electrical tube furnace with Kanthal Super heating elements and an alumina reaction tube (70 mm inner diameter) was used. On one end of the tube, an internally water-cooled quenching chamber seals the tube with a Viton O-ring, and on the opposite end, the tube was sealed with a water-cooled aluminum cap with a sealed borosilicate glass window. The specimen was placed on a graphite carriage that transports the specimen in and out of the hot zone of the furnace, guided on a graphite track. A water-cooled pushrod, sealed with a shaft packing, was connected to the graphite carriage, which in turn is fastened to a screw drive that enables the precise positioning and movement of the specimen for controlled heating and cooling rates. A Eurotherm controller maintains the target furnace temperature with an even temperature zone, defined as ±2 K from the target temperature. The controller thermocouple was a type B thermocouple (mounted in the wall of the furnace), which was positioned in the hot zone of the furnace outside of the reaction tube. Type C thermocouple with 1% accuracy was mounted axially in the pushrod with the thermocouple tip inside the carriage body positioned directly under the specimen substrate. After pushing the sample holder, the furnace was evacuated and backfilled with pure argon (Ar) at least three times. High-purity Ar gas (>99.999%) was used with a flow rate of 0.1 L/min using a Bronkhorst mass flow meter for this purpose. After refilling, furnace hearing was started on a EuroTherm controller at a controlled rate of 2 K/min. A Leica V-LUX3 digital camera with full high-definition video (1920 × 1080 progressively displayed pixels) capability at 30 frames per second was mounted with a view into the borosilicate glass window at the end of the furnace. Video recordings were initiated when the specimen melts at ∼1813 K. The melting occurred over a temperature range and was not instantaneous. The images were extracted from the video frames. The contact angle measurement was then analyzed using images with low-bond axisymmetric drop shape analysis (LB-ADSA),^40^ which involved fitting the Young–Laplace equation to image data. The contact angles were obtained from the images captured during the experiment. The droplet diameters were in the order of 10 mm; see Figure S2 for details. In the experiment, the metal transitioned into a molten state within a specified temperature range and stabilized at ∼1813 K (∼1540 °C). The contact angle was initially measured at the melting point, at a time denoted @*t*_0_, capturing the immediate formation and behavior of the droplet. The contact angle was measured subsequently five times taken between 50 and 150 min postmelting. The final contact angle was determined at this later stage of the measurements, denoted @*t*_c_, as the average value with a maximum deviation of ±3° in the subsequent measured data (Figure S3a). The error bar of each measurement was estimated to be at most ±5°. This image-based measurement approach ensured that the reported contact angles were derived from a stable and consistent visual record.

The contact angles for the tin–bismuth/oxide systems consider a liquid metal alloy with 40 wt % bismuth and were measured in open air rather than in a furnace. Using a Sn–Bi wire sourced from Chip Quik Inc. (Ontario, Canada), the alloy was heated to about 473 K (200 °C) until a droplet formed. This droplet was then placed on the oxide substrate. A camera captured the image of the droplet in situ, and the DropSnake method was later used for image analysis, defining the contour of the drop as a versatile B-spline curve.^41^ The technique analyzed the fitted curvatures to obtain measurements of contact angles. The measurements were taken for two specific angles: one when the left side of the droplet contacts the substrate and another when the right side touches. This helped to understand the droplet’s interaction with the oxide surface. Experiments were conducted in open air rather than under Ar gas conditions to avoid the complexities associated with setting up a controlled inert atmosphere for low melting point materials. In this study, stringent oxidation protection was not considered for the low melting of this metal alloy. Open-air conditions simplified the experimental setup and were sufficient for measurement of the wettability of the tin–bismuth alloy. Surface oxygen can influence the wetting behavior; however, the small scale of the SnBi experiments and the associated complexities of modifying the setup made inert gas usage impractical for these measurements. To minimize the influence of surface oxides, samples were prepared for each experiment and measurements were taken immediately after droplet formation. The decision to perform these measurements in open air was made after careful consideration of these limitations, and consistency across the experiments ensured that any potential influence of surface oxides remained uniform. However, for future studies, a more carefully designed setup should be planned to enable measurements under inert gas conditions, ensuring further control over the experimental parameters and minimizing the influence of surface oxidation.

## Results and Discussion

### Crystal Structures of Oxides

The crystal structures of the oxides are relaxed, and the electronic structures are computed with the PBEsol exchange-correlation functional. The lattice parameters are presented in Table 1. One can observe that the theoretically determined crystal structures agree very well with our RT-XRD analysis (Figures S4–S13) as well with earlier published experimental data. Deviation of the lattice parameters between different reported measurements is small for these oxides, also in early publications, and the data are thus well-established. We therefore choose to present only two representative values for each lattice parameter: one from the handbook by Samsonov^42^ and another from the Landolt–Börnstein database.^43^ Furthermore, the table presents the calculated Γ-point direct band gap energies employing the hybrid functional with 30% exact exchange in order to generate proper gap values. These energies are compared to available and reasonable experimental data from the two main literature references. One notices a significant deviation of the reported energies between different measurements is observed, which probably can be ascribed to variations in the crystal quality regarding defect inclusion. Hence, values of the band gap energy are not fully established with high precision.

**Table 1:** Lattice Parameters *a*, *b*, *c*, and β for the Seven Refractory Oxides Determined with the PBEsol Exchange-Correlation Functional and the RT-XRD Analysis^a^

oxide	DFT	RT-XRD	literature
Al_2_O_3_; *R*3̅*c*			
*a* (Å)	5.137	5.131	5.128;^42^ 5.13^43^
β (deg)	55.34	55.28	55.28;^42^ 55.27^43^
*E*_g,Γ_^dir^ (eV)	8.7		2.5; 3.59;^42^ 7; 9; 9.26^43^
CaO; *Fm*3̅*m*			
*a* (Å)	4.762		4.799;^42^ 4.810^43^
*E*_g,Γ_^dir^ (eV)	6.8		5.59;^42^ 7.085; 7.7^43^
MgO; *Fm*3̅*m*			
*a* (Å)	4.211	4.2222	4.20;^42^ 4.212^43^
*E*_g,Γ_^dir^ (eV)	6.9		7.29;^42^ 5.13; 7.833^43^
SiO_2_; *P*3_1_21			
*a* (Å)	4.905	4.9191	4.913;^42^ 4.9134^43^
*c* (Å)	5.405	5.4105	5.405;^42^ 5.4046^43^
*E*_g,Γ_^dir^ (eV)	8.5		9.1; 9.43^43^
SiO_2_; *Fd*3̅*m*			
*a* (Å)	7.360		7.1487;^44^ 7.1393^43^
*E*_g,Γ_^dir^ (eV)	7.7		
ZrO_2_; *P*2_1_/*c*			
*a* (Å)	5.126	5.1487	5.17;^42^ 5.127^43^
*b* (Å)	5.213	5.2098	5.26;^42^ 5.202^43^
*c* (Å)	5.292	5.3149	5.30;^42^ 5.31^43^
β (deg)	99.57	99.204	99.90;^42^ 99.18^43^
*E*_g,Γ_^dir^ (eV)	6.0		2.0;^42^ 5.83; 7.09^43^
ZrO_2_; *P*4_2_/*nmc*			
*a* (Å)	3.584		3.578^43^
*c* (Å)	5.175		5.19^43^
*E*_g,Γ_^dir^ (eV)	6.1		0.74; 3.3^43^

a The direct band gap energy *E*_g,Γ_^dir^ refers to the Γ-point obtained with the hybrid functional HSE. The corresponding experimental energies represent typically the optical gaps.

Alumina is the main refractory component in the nozzle walls for steelmaking processes. The compound is thermodynamically stable in the α-phase, which is a rhombohedral structure (space group #167; *R*3̅*c*). The RT-XRD determined lattice parameters of Al_2_O_3_ confirm a good crystalline quality. The compound is often presented based on the three times larger hexagonal unit cell, and the RT-XRD data are then *a* = 4.761 Å and *c*/*a* = 2.730. The corresponding DFT values are *a* = 4.771 Å and *c*/*a* = 2.726. There are two types of bonds: each Al atom has three bonds with length δ_b_(Al–O) = 1.86 Å and three with length δ_b_(Al–O) = 1.97 Å. According to the empirically measured covalent atomic radii by Slater,^45^ the Al atom has a radius of 1.25 Å and the O atom has a radius of 0.60 Å; thus, one could expect a smallest bond length in the order of 1.85 Å. The band gap energy of 8.7 eV from the DFT calculations is in line with the recent measurements,^43^ while older experiments underestimate the gap quite significantly.^42^

At room temperature, and also at 1823 K, calcia crystallizes in a face-centered cubic (“cub”) phase, more precisely the rock salt structure (space group #225; *Fm*3̅*m*). Each atom in CaO is 6-fold-coordinated with a bond length of δ_*b*_(Ca–O) = 2.38 Å. The calculated band gap energy at the Γ-point is 6.8 eV, which is on the same order as the other refractory oxides. However, our RT-XRD analysis of CaO powder does not support single crystalline cubic CaO. Instead, the powder is composed of a mixture of phases with a strong weight fraction of hexagonal calcium hydroxide. That is not surprising as calcia is exothermally hygroscopic under ambient humidity conditions.^46^ The experimentally determined lattice parameters are *a* = 3.592 Å and *c*/*a* = 1.371, which are consistent with our theoretical data for trigonal Ca(OH)_2_ with space group *P*3®*m*1: 3.555 Å and 1.334, respectively. The powder also contains calcium carbonate, CaCO_3_, which exists in various phases with formation energy <0.02 eV/atom above hull.^47^ The most stable polymorph at room temperature is calcite, which has a similar structure as Al_2_O_3_. DFT yields *a* = 6.308 Å and β = 46.57°, which agree with data from several reports.^43^ The RT-XRD detects, however, a mixture of an orthorhombic and a monoclinic structure, which suggests inclusion of aragonite. In addition, the RT-XRD reveals a hexagonal phase of CaO; see Figure S5. The structure has, to the best of our knowledge, not been experimentally observed before. The lattice constants are *a* = 3.945 Å and *c*/*a* = 1.158. DFT calculation of hexagonal CaO (#194; *P*6_3_/*mmc*) seems to support that structure with *a* = 3.952 Å and *c*/*a* = 1.199, despite a significantly larger lattice constant in the [0001] direction. The DFT formation enthalpy of this hexagonal phase is, however, 90 meV/atom higher than that of its cubic counterpart, indicating that the polymorph is unlikely to be formed as a single-crystal high-quality substrate.

Magnesia is thermodynamically stable in a rock salt structure similar to that of cubic calcia. Also, the Γ-point gap energies of the two cubic compounds are very similar: 6.9 eV for MgO. However, since the Mg atom is smaller than Ca, the corresponding bond lengths are smaller: δ_b_(Mg–O) = 2.11 Å. Actually, these bond lengths are closer to those of Al_2_O_3_. Both Mg and Al are period-3 elements with rather similar atomic radii, although Mg is ∼20% larger. The obvious difference between MgO and Al_2_O_3_ is the cation valence configurations (group IIA versus group IIIA), which thereby form different covalent bond symmetries by the octet rule. In addition to the cubic MgO structure, the XRD measurement also identifies contribution from trigonal Mg(OH)_2_, expected to be present on the surfaces of powder particles.

Silica is composed of two most abundant elements in the Earth’s crust, and the quartz family of silica exists in different varieties. At room temperature, α-SiO_2_ is the most stable phase, whose structure is trigonal (“tri”; #152; *P*3_1_21). Each Si has four bonds with δ_b_(Si–O) of 1.62 Å. At higher temperatures, this SiO_2_(tri) phase is easily and abruptly transformed to other phases, and at 1823 K it is expected to become face-centered cubic β-cristobalite. The computed formation energy of ideal β-cristobalite (#227; *Fd*3̅*m*) is 19 meV/atom above the convex hull. This structure is notably larger than the trigonal counterpart, but this fact is not reflected in the bond distance (1.59 Å) nor is it explained by any change in the coordination number. Experimental analysis has identified the high-temperature compound as a *Fd*3̅*m* structure, however, with partially occupied Wyckoff sites.^44^ An alternative phase is a distorted structure that lowers the symmetry to cubic *P*2_1_3.^43^ One should not expect perfect agreement between computed and measured lattice parameters. The calculated band gap energy 8.5 eV of SiO_2_(tri) agrees rather well with experimental findings, which mostly are in the range of 8.5–9.5 eV. SiO_2_(cub) has ∼1 eV smaller gap.

Zirconia is often listed as a high-temperature κ dielectric material. The compound crystallizes in a monoclinic structure at room temperature (“mono”; #14; *P*2_1_/*c*). Due to low crystalline symmetry, each Zn has different bond distances to its surrounding O atoms: 2.05, 2.06, 2.15, 2.15, 2.17, 2.23, and 2.25 Å. The high-temperature phase of ZrO_2_ is however tetragonal (“tetra”; #137; *P*4_2_/*nmc*). The DFT formation enthalpy of ZrO_2_(tetra), neglecting temperature effects, is 26 meV larger than that of ZrO_2_(mono). The two phases have very similar band gap energies: 6.1 versus 6.0 eV. In ZrO_2_(tetra), each Zr atom has four bonds with δ_b_(Zr–O) = 2.07 Å and four bonds with 2.36 Å. From the covalent atomic radii,^45^ one would expect that the bond distances should be around 2.15 Å.

### Dielectric Response Functions

The frequency-dependent dielectric response function, or the relative permittivity, is a fundamental property to describe the electric displacement field resulting from an applied electric field. It is also the main optical characteristic of a material, often represented by its dielectric constant(s). Having established the crystalline structures of the oxides, the complex dielectric functions are calculated according to eqs 1–3. The resulting static ε_0_ and high-frequency ε_∞_ dielectric constants (Table 2) are determined at zero frequency by, respectively, including and disregarding the contribution from optical vibrations. This approach is somewhat different from the experimental determination. The static dielectric constant is typically measured at very low frequency, so that it should be comparable to the DFT values. The high-frequency dielectric constant is however experimentally resolved at a frequency corresponding to an energy that should be sufficiently below the optical gap. Since the dielectric function in that region is increasing as a function of frequency, one could expect a somewhat larger measured value compared to the theory; however, the deviation is typically not large, often between 0.1 and 0.3.

**Table 2:** Static ε_0_ and High-Frequency ε_∞_ Dielectric Constants of the Oxides and in the Three Cartesian Directions, i.e., the Diagonal Components of the 3 × 3 Tensor

	DFT			expt.
oxide	*xx*	*yy*	*zz*	*xx*/*zz*
Al_2_O_3_				
ε_0_	9.3	*xx*	11.4	10.5–12; 12.3;^42^ 9.34/11.54^43^
ε_∞_	3.0	*xx*	3.0	
CaO				
ε_0_	15.3	*xx*	*xx*	3.00–0.10; 11.8;^42^ 11.96^43^
ε_∞_	3.4	*xx*	*xx*	3.33; 3.385^43^
MgO				
ε_0_	9.9	*xx*	*xx*	3.2–3.1; 8–10.5;^42^ 9.95^43^
ε_∞_	2.9	*xx*	*xx*	
SiO_2_(tri)				
ε_0_	4.5	*xx*	4.7	3.5–4.1; 4.34/4.27;^42^ 4.42/4.60^43^
ε_∞_	2.4	*xx*	2.4	
SiO_2_(cub)				
ε_0_	3.8	*xx*	*xx*	
ε_∞_	2.0	*xx*	*xx*	
ZrO_2_(mono)				
ε_0_	20.5	22.4	16.2	12.5; 23^43^
ε_∞_	4.6	4.6	4.3	
ZrO_2_(tetra)				
ε_0_	63.5	*xx*	18.9	
ε_∞_	4.9	*xx*	4.5	

From Table 2, one can observe that all of the considered oxides exhibit high-frequency dielectric constants between 2.0 and 4.9, where SiO_2_(cub) has the lowest value and ZrO_2_(tetra) has the highest value. The spatial anisotropy of the high-frequency dielectric constants is small, at most ∼7% (for ZrO_2_) and zero by symmetry, which is a reason for the cubic phases. Moreover, the two phases of SiO_2_ have similar constants, and this is also true for the two phases of ZrO_2_. The static dielectric constants are however more different between the oxides. These values are also more anisotropic, as it depends on the local bond symmetries. Here, the contribution from the optical phonon vibration is only ∼2 for the two SiO_2_ phases, whereas it is ∼6–8 for Al_2_O_3_ and MgO, ∼12 for CaO, and as much as ∼12–60 for ZrO_2_. The theoretical data agree with experimental results for the most common and well-studied oxides, i.e., Al_2_O_3_, MgO, and SiO_2_(tri), while the data for high-κ ZrO_2_(mono) seem not to be fully established. One can notice that the tetragonal and monoclinic phases of ZrO_2_ have similar high-frequency dielectric constants, but at low frequencies, their dielectric functions diverge by as much as approximately three times in the *xy* plane. Notable is also the high spatial anisotropy of the static dielectric constant for high-temperature phase ZrO_2_(tetra).

**Figure 2:**
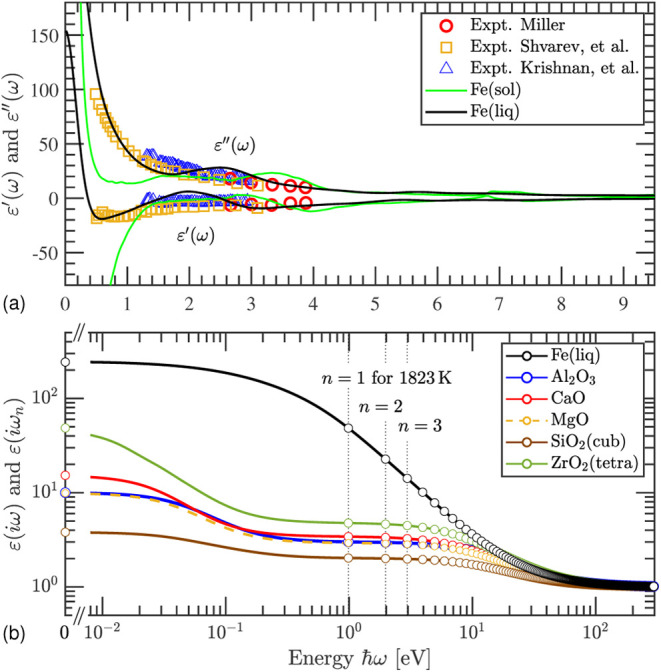
(a) Dielectric function of the modeled liquid Fe (Fe(liq); black lines), compared with the solid Fe at room temperature (Fe(sol); green lines) and available experimental data (marks) from refs (48−50). (b) Dielectric functions of liquid Fe and the refractory oxides elaborate on the imaginary axis. Here, the circles indicate data at Matsubara frequencies ω_*n*_. At zero frequency (i.e., ω = ω_0_ = 0), the data equal ∼244 for Fe(liq) and the average static dielectric constants ε_0_ for the oxides, which can be obtained also from Table 2.

The complex dielectric function of liquid iron, Fe(liq), is modeled for the temperature of 1823 K with an ideal crystalline structure. We verify that the model is reasonable by comparing the resulting spectrum with the corresponding spectrum for room-temperature solid iron, Fe(sol), as well as by comparing it with available measured dielectric responses; see Figure 2a. First, there are clear differences between the dielectric functions of Fe(sol) and Fe(liq). The reason is due to the change in volume, where Fe(liq) has 11% lower density and also due to different magnetic configurations. Here, our calculated ferromagnetic Fe(sol) has an intrinsic magnetic moment of 2.6 μ_B_/atom, which is close to the measured ∼2.4 μ_B_/atom, whereas Fe(liq) is paramagnetic. This affects the dielectric dispersion especially for energies below 1.5 eV and also in the region between about 2 and 4 eV. Second, the dielectric function of Fe(liq) is compared with experimental reports by Miller,^48^ Shvarev et al.,^49^ and Krishnan et al.^50^ The three measured spectra are complementary and overlapping, covering an energy region from about 0.5 to 4.0 eV. In this region, our modeled dielectric function of Fe(liq) agrees fairly well with the measured data. Moreover, the theoretical response function goes asymptotically correctly to one at high energies. In addition, based on the dielectric function and using a similar model as eq 5, our calculated surface tension of Fe(liq) is γ_1_ = 1.843 J/m^2^, which agrees well with the measured data between 1.83 and 1.92 J/m^2^ with the adopted mean values of 1.85, 1.88, or 1.90 J/m^2^.^51,52^ We therefore anticipate that our model for describing the dielectric response of liquid iron is reasonable for exploring the Casimir–Lifshitz dispersion forces of the Fe(liq)/oxide systems.

The dielectric response functions are elaborated on the imaginary axis according to eq 4, and the resulting spectra for Fe(liq) and the high-temperature phases of the oxides are presented in Figure 2b. The circles represent the data for Matsubara’s frequencies for *T* = 1823 K. The liquid metal has a much larger dielectric function than the oxides at low frequencies due to the Drude contribution from the (almost) free electrons. On the other hand, the metal does not exhibit the oxides’ declining dispersion at ∼0.01–0.5 eV, which is a consequence of the lattice dynamics not playing an important role at energies higher than ∼1 eV. The first Matsubara frequency (i.e., *n* = 0) yields the static dielectric constant ε_0_. For the second Matsubara frequency (i.e., *n* = 1), the oxides’ dielectric functions are close to their respective high-frequency dielectric constants ε_∞_. In the energy region up to some tens of eV, the spectrum of ZrO_2_(tetra) is the largest of the considered oxides, and the spectrum of SiO_2_(cub) is the smallest, whereas Al_2_O_3_, MgO, and CaO have rather similar spectra. The spectra decline smoothly even further at energies that correspond to the respective band gap energies. At very high energies, the spectra of the oxides go, just like that of Fe(liq), asymptotically to one.

The dielectric response functions of the oxides and liquids are essential representations for describing the interaction under high-temperature conditions, which in turn also influence material factors such as the thermal stability and wettability of the substrates by the liquids.

### Wettability of Liquid-on-Oxide

The strength of the cross-interface interaction in a liquid–oxide system is described by the work of adhesion *W*, which is often represented by Hamaker constant *A*_123_. We theoretically determine the Hamaker constants of the Fe(liq)//oxide systems directly from the dielectric response functions according to eq 7 and at the temperature *T* = 1823 K. In Table 3, it is clear that ZrO_2_ has the largest Hamaker constant of these refractory oxides. The constant of SiO_2_ is the smallest, whereas Al_2_O_3_, MgO, and CaO have similar Hamaker constants. This is consistent with the strengths of the oxides’ dielectric response functions (Figure 2b). Representing the liquid–substrate interaction only by the Hamaker constant is, however, not unambiguous. It is a parameter useful for describing, for example, forces between macroscopic objects at sufficiently large distances, but the three-layer model in eq 5 is not applicable for an infinitely small interlayer thickness. The reason is that the physical quantity, i.e., the work of adhesion, is proportional to *A*_123_/*d*_0_^2^ and thus involves a finite size of the auxiliary gap. As a consequence, an incorrect determination of the Hamaker constant could generate a correct value of *W* if also the size of *d*_0_ is adjusted. However, the Hamaker constant could not be utilized as a parameter to describe forces as a function of the distance. Hence, a consistent determination of *d*_0_ is required, accompanying the presentation of *A*_123_ in order to properly describe the wettability.

**Table 3:** Hamaker Constants *A*_123_, Contact Angles θ, Interfacial Distances *d*_0_, Works of Adhesion *W*, and Corresponding Forces *F*_0_ per Unit Area for Fe(liq)-on-Oxide Systems^a^

		θ (arc deg)^c^					
oxide	*A*_123_ (10^–19^ J)	@*t*_0_	@*t*_c_	literature	*d*_0_ (nm)	*W* (J/m^2^)	*F*_0_ (10^9^ N/m^2^)
Al_2_O_3_	2.75	134	136	135; 144;^53^^,^^b^ 126.9;^55^ 132;^56^ 139; 141;^42^ 118–129^54^^,^^b^	0.119	0.52	8.71
CaO	2.76	106	104	121;^53^ 132;^42^ 104–114^54^^,^^b^	0.072	1.40	38.56
MgO	2.57	124	122	128;^53^ 133.5;^55^ 123; 130;^42^ 96–122^54^^,^^b^	0.089	0.87	19.53
SiO_2_(tri)	2.14	117	116	110;^53^ 105–135;^57^ 108;^42^ 116–128^54^^,^^b^	0.074	1.03	27.95
SiO_2_(cub)	1.72				0.066	1.03	31.14
ZrO_2_(mono)	3.26	152	143	119; 122;^53^ 92; 102; 111;^42^ 105–120^54^^,^^b^	0.153	0.37	4.86
ZrO_2_(tetra)	3.39				0.156	0.37	4.77

a The measured contact angles θ are presented at the initial time when reaching *T* = 1823 K (column denoted @*t*_0_), and when measuring at the later stage (@*t*_c_); the latter is our confirmed average value of the contact angles with a maximum deviation of ±3°. Cubic SiO_2_ and tetragonal ZrO_2_ are expected to be the respective high-temperature phases. The results are compared to experimental contact angles in the literature.

b Experimental data at *T* = 1873 K.

c The crystal structures are not experimentally determined at high temperatures, however, cubic SiO_2_ and tetragonal ZrO_2_ are expected to be the respective high-temperature phases.

**Figure 3:**
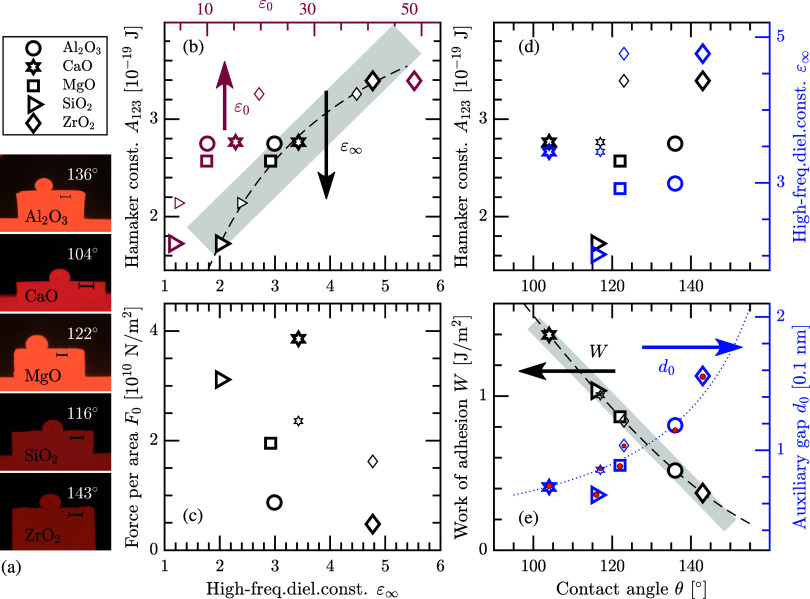
(a) Experimentally determined contact angles of Fe(liq)-on-oxides, all at *T* = 1823 K and under argon flow conditions. The black scale bars indicate 5 mm. Panels (b)–(d) describe property relations for systems with Al_2_O_3_ (circles), CaO (hexagrams), MgO (squares), SiO_2_ (triangles), and ZrO_2_ (diamond symbols). Dashed and dotted lines represent fitted curvatures, and gray areas are guides for the eye to indicate an almost linear relation. The smaller marks in panel (b) are the corresponding results for the room-temperature phases of SiO_2_ and ZrO_2_, whereas the smaller marks in panels (c)–(e) are the results for CaO and ZrO_2_(tetra) when using the average values of their contact angles θ_ave_, i.e., including earlier published data. (b) Hamaker constant *A*_123_ versus the high-frequency ε_∞_ (bottom axis) and static ε_0_ (top axis) dielectric constant. (c) Force *F*_0_ per area versus the high-frequency dielectric constant. (d) Hamaker constant (left axis) and high-frequency dielectric constant (right axis) versus the contact angle θ. (e) Work of adhesion *W* (left axis) and the interlayer gap distance *d*_0_ (right axis) as functions of the contact angle. The red filled circles represent an approximated expression, utilizing the fit of the Hamaker constant in panel (b), as generated by eq 9.

In the experimental investigation of the wettability of melted iron on different oxide surfaces, a distinct variation in the contact angles is observed from the sessile drop measurements (Figure 3a and Table 3). Here, the cubic structure of SiO_2_ and the tetragonal structure of ZrO_2_ are considered as high-temperature phases. The results for the five Fe(liq)/oxides, a direct indicator of their wettability at *T* = 1823 K, range from moderate to low wetting surfaces. Calcia presents a contact angle of 104°, suggesting a moderate degree of wettability. A slightly higher contact angle of 116° is noted for silica, indicating a reduced wettability. This trend of increasing contact angles continues with magnesia, which exhibits a contact angle of 122°, and alumina that shows a further increased contact angle of 136°, implying an even lower wettability. The most significant resistance to wetting is demonstrated by zirconia, with a contact angle of initially 152°, which changes over time to be stabilized at 143°. For water, a surface is described as superhydrophobic if the water/substrate system has a static contact angle that exceeds 150°.^19^ Our measured contact angle for Fe(liq)/ZrO_2_ is close to that very low degree of wettability; however, earlier reported data indicate a smaller angle of around 120°.^53,54^

Experimental approaches for measuring contact angles at high temperatures, e.g., 1823 K, often overlook the nuanced significance of accurately considering and treating competing phases at the liquid–oxide interface. The potential formation of intermediate phases, such as FeAl_2_O_4_ in the Fe(liq)/Al_2_O_3_ system, could complicate a precise measurement of the contact angles. This uncertainty arises from the ambiguity in determining the thermodynamic stability of an ideal interface contrary to chemical reaction or adsorption, which questions whether the work of adhesion with its auxiliary gap relates to the Fe(liq)/Al_2_O_3_ interface or a Fe(liq)/FeAl_2_O_4_/Al_2_O_3_ interface. However, an ultrathin modified surface layer is not expected to have a decisive significance for the cross-interface interactions. Historical sessile drop experiments have reported varying contact angles for Fe/Al_2_O_3_,^42,54^ perhaps indicating that an intermediate phase of a thicker size influences the measurements. However, our result θ = 136° for alumina is consistent with the most recent data in the literature.^53,55,56^ The complexity with competing phases extends to other systems as well. For instance, calcia is hygroscopic and Ca(OH)_2_ is easily formed. This should, however, be less problematic for the measurement of Fe(liq)/CaO because H_2_O is expected to evaporate above *T* ≈ 785 K (512 °C) and the substrate will transform to CaO. Similarly, contamination of CaCO_3_ will decompose into CaO and CO_2_ beyond its calcination temperature ∼1023 K (750 °C). Our measured contact angle of calcia, θ = 104°, is, however, smaller than earlier published data, although those data span a quite large region of angles. It might be that a thin film of liquid calcite is present at the Fe(liq)/CaO interface due to the cross-interface pressure of almost 40 GPa; such effect is supported by phase diagram from Ivanov et al.^58^ A perhaps more plausible explanation is that carbon from the CO_2_ vapor contaminates molten iron with the release of O_2_. Support for such effect is that the wettability of alumina is higher by carbon-containing molten iron than by pure liquid iron.^42^ The CaO samples used in our experiment may differ from those referred to in the literature, especially in terms of purity, density, and crystalline structure. A related effect to consider is the possibility of oxygen adsorption on the surface of the metal droplet.^56^ If the concentration of oxygen at the surface is high, then there could be diffusion of oxygen from the surface to the bulk. Such an oxidation process may contribute to the change in the contact angle with respect to time. Further, in the Fe(liq)/MgO system, the high-temperature interactions could lead to the formation of phases like MgFe_2_O_4_, which would alter the interfacial characteristics and, consequently, the contact angles. Similarly, in the Fe(liq)/SiO_2_ system, the presence of iron silicates at the interface may impact the wettability and work of adhesion. The Fe(liq)/ZrO_2_ system also presents similar challenges, and in addition, the substrate should be able to transform from the monoclinic to tetragonal phase as the substrate is heating up above 1443 K (1170 °C). This could be a possible reason for the notably decrease in the contact angle over time. The presence of impurities and increased surface roughness may increase the contact angles by restricting the spread of liquid metal across the ZrO_2_ substrate. Another issue is associated with the measurement conditions. The choice of applied gas in the furnace, in our case argon, as well as its pressure will affect especially the liquid surface tension. For low and moderate gas pressures, the effect should be negligible. At high applied gas pressure at high temperatures, one could expect some further amorphorization of SiO_2_ and an inclusion of the β-quartz phase.

Overall, however, our measured contact angles of the Fe(liq)/oxide systems agree fairly well within the range of published data in the literature (Table 3 and Figure S3b). Nonetheless, for Fe(liq)/CaO and Fe(liq)/ZrO_2_, we will consider also the average value of their contact angles, including the earlier reasonable experimental data: θ_ave_ = 117 and 123°, respectively.

There could be several sources for the deviation of the reported contact angles; see also Figure S3b. For example, Kapilashrami et al. discuss that their observed rapid decline in contact angle for liquid Fe (with inclusion of 0.0004% Mn) on the SiO_2_ surface, and with 1.5 × 10^–5^ Pa O_2_ pressure, could be due to the formation of Fe_2_SiO_4_ if oxygen dissolved in the iron melt exceeds a concentration of 0.1 wt %. Humenik and Kingery^59^ (ref 520 in Samsonov^42^ and ref 27 in Nakashima et al.^53^) reported for Fe(liq)/MgO and Fe(liq)/ZrO_2_ some 10° larger contact angles in He gas compared to vacuum, and for ZrO_2_ even ∼10° larger if using H_2_. Working gas pressure was about 7 × 10^–4^ Pa. Their surface tensions for iron were, however, obtained only in the hydrogen and helium environment. The MgO sample had minor constituents of SiO_2_, CaO, Al_2_O_3_, and Fe_2_O_3_. ZrO_2_ was stabilized with ∼95% ZrO + HfO. Also for Al_2_O_3_ (with minor constituents of SiO_2_ and Fe_2_O_3_), the use of H_2_ instead of He increased the angle by ∼10°. Moreover, they found that the work of adhesion of Fe(liq)/Al_2_O_3_ remains nearly constant for electrolytic iron, with slightly larger values for Armco iron. Their drop dimensions for determining the contact angles were estimated to deviate by ±2% and ±5% for two different analysis methods. The same mean values of the angle were however obtained by both methods.

The Casimir–Lifshitz free energy and the corresponding Hamaker constant depend explicitly on the dielectric response functions of the materials for the liquid//substrate layers. In Figure 3b, we present the Hamaker constants of Fe(liq)//oxides versus the high-frequency ε_∞_ (bottom axis) and static ε_0_ (top axis) dielectric constants of the oxides. The Hamaker constant increases almost linearly with the size of the high-frequency dielectric constant. A simple linear regression analysis yields (0.820 + 0.555ε_∞_) × 10^–19^ J with the coefficient of determination *R*^2^ = 0.928; gray area in the figure. However, a more accurate relation is obtained by observing that *A*_123_ in eq 7 goes roughly proportional to (ε_3_ – 1)/(ε_3_ + 1) for systems with ε_1_ ≫ ε_2_ and ε_3_ ≳ ε_2_ = 1. Assume now that the difference in the oxides’ response behaviors is dominated by their respective value of the high-frequency dielectric constant. Then, the relation should be
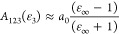
9where *a*_0_ is a nonphysical quantity that in principal should describe the sum over Matsubara frequencies and the integration over *z* in eq 7. In eq 9, however, *a*_0_ is treated as a constant fitting parameter to the expression. A regression analysis of eq 9, employing ε_∞_ from Table 2, results in *a*_0_ = 5.15 × 10^–19^ J with *R*^2^ = 0.966 for the Fe(liq)//oxide systems. This plain and manageable relation describes the trend of the Hamaker constant surprisingly well; see the dashed black line in Figure 3b. The relation equals correctly zero if the dielectric constant of the substrate would be one. Furthermore, we determine the value of *a*_0_ with only the high-temperature phases of the oxides. However, since the relation should be applicable for any phase, we display also the data for SiO_2_(tri) and ZrO_2_(mono), represented by the smaller marks in the figure, demonstrating the good behavior of eq 9.

From Figure 3b, one might think that there should be a similar relation also for the Hamaker constant versus the static dielectric constant, ε_0_, of the oxides. However, this is not applicable in our case, especially for ZrO_2_(tetra). The static dielectric constants are less important at high temperatures and for two layers that are in close contact with each other.^60^ This is clear when comparing the results for ZrO_2_(mono) and ZrO_2_(tetra). The two phases have similar *A*_123_ and comparable ε_∞_, but their ε_0_ values differ by a factor of about 2.5. In our case, ε_0_ contributes only for the first Matsubara frequency (*n* = 0), whereas the spectrum of the dielectric function relates to ε_∞_ in a much larger energy region. Hence, the main contribution to the Casimir–Lifshitz forces is, for the present systems and temperature, the electronic transitions (i.e., eq 1).

Wettability and stiction depend on the force between the liquid and the substrate, which are calculated by the derivative of the work with respect to the distance determined at equilibrium distance *d*_0_. The resulting expression *F*_0_ = *A*_123_/6π*d*_0_^3^ describes the force per area (alternatively, the pressure), where a positive value implies attraction. We do not find any straightforward trend between the force and dielectric constant (Figure 3c). Since our measured contact angles for calcia and zirconia deviate from the literature data, we present with small marks in the figure also the results for the average values of their contact angles. Still, there is no clear trend. This may seem surprising because there exists a good relation between the Hamaker constant and the high-frequency dielectric constant. However, as a consequence of the definition of the Hamaker constant (eq 7), *A*_123_ is independent of the auxiliary gap *d*_0_; this is evident from the right side of the equation. On the other hand, the forces (as well as the work) depend on the gap *d*_0_ through the exponential function exp(−2κ_2_*d*_0_) in eq 5. The work of adhesion is proportional to *A*_123_/*d*_0_^2^, and the force is proportional to *A*_123_/*d*_0_^3^. Since there exists a relation between *A*_123_ and ε_∞_, there must be a clear correlation between *d*_0_ and ε_∞_ in order to have a trend between *F* and ε_∞_. However, the correlation between *d*_0_ and ε_∞_ is not obvious. For example, one can notice that zirconia has the smallest force (and smallest *W*; Table 3) of all five oxides, despite having the largest dielectric constant, which in turn implies the largest Hamaker constant according to Figure 3b. Thus, the largest *A*_123_ combined with the smallest *W* ∝ *A*_123_/*d*_0_^2^ implies that *d*_0_ must be much larger than the gap of most other oxides, which is also seen in Table 3. The explanation is that the large gap weakens the cross-gap interactions. Alumina and calcia have comparable high-frequency dielectric constants and Hamaker constants, but their forces and gaps deviate significantly. Hence, one cannot draw conclusions about the wettability or stiction based only on the size of the Hamaker constant. Instead, one also has to analyze the contact angle in relation to the auxiliary gap.

In order to model the wettability of liquid–substrate systems, one should first describe the relation between the material properties and contact angle θ. We do not find any straight relation between the Hamaker constant and the contact angle (Figure 3d, left axis) or between the dielectric constant and the contact angle (right axis in the figure). That is true also when considering the average values of the measured contact angles θ_ave_ for CaO and ZrO_2_; the smaller marks in the figure. Hence, one has to incorporate the auxiliary gap *d*_0_ in the description of the cross-interface interaction, i.e., by modeling *W* versus θ together with a description of *d*_0_ versus θ.

One can in Table 3 observe that the work of adhesion is declining with an increasing contact angle. This is expected because the energy to separate the liquid droplet from the surface should be smaller for lower wettability. The dependence is almost linear with *W* ≈ 4.097 – 0.026θ J/m^2^ (*R*^2^ = 0.996); gray area in Figure 3e. However, the correct relation is *W* = γ_1_·(cos θ + 1), which is displayed by the black dashed line in the figure. This relation goes to zero as the contact angle reaches 180°, i.e., for completely nonwetting when the droplet is not attracted to the surface. Moreover, the maximum theoretical value of the work of adhesion is 2γ_1_ = 3.68 J/m^2^, which would occur at 0° when the liquid completely wets the substrate and forms a thin uniform layer.

As discussed, the work of adhesion depends on the Hamaker constant, which is not an unambiguous material parameter unless it is accompanied by a consistent determination of the auxiliary gap. The gap is even more important when describing the cross-interface force as it is inversely proportional to *d*_0_^3^. It is therefore crucial to understand and correctly model *d*_0_ when describing interfacial properties such as stiction of molten iron on the refractory nozzle wall, particle clogging of the nozzle, or oxide particle–particle agglomeration in the melt. Here, *d*_0_ is described as an auxiliary distance because it depends on how the interface is described by the three-layer model (eq 7) and also because *d*_0_ is in the order of 0.1 nm (1 Å). The diameters of atoms are typically 0.1–0.3 nm (e.g., the Ar atom has a diameter of ∼0.15 nm),^45^ and *d*_0_ can therefore not be associated with a macroscopic layer of physical air or an argon gas. Certainly, sole atoms could penetrate into the interfacial layer; however, that would have negligible impact on the Casimir–Lifshitz forces. Also, treating this effect would imply modeling of the atomic polarizability as well as including surface and interface relaxation of Fe(liq) and the oxides and is beyond the present macroscopic model of the material systems. Instead, it is an uttermost advantage with the present methodology to be able to accurately explore the cross-interface interactions with a macroscopic description of the layered systems combined with an atomistic calculation of bulk material properties.

With the computed Hamaker constants (Table 3), it is clear that the auxiliary gap typically increases when the wettability becomes lower, i.e., for larger contact angles; see blue marks and right axis in Figure 3e. This is reasonable because a weaker interfacial interaction should yield a larger gap. Even though it is preferable with an explicit calculation of the Hamaker constant and its corresponding auxiliary gap, it would be useful if one could predict *d*_0_ and θ for any new oxide compound or related alloys. From eq 8, it is clear that the gap depends on the square root of the Hamaker constant. Since *A*_123_ does not deviate too much between the oxides, a very rough estimate would be to use a constant value of it (*A*_123_) and *d*_0_ ≈ [*A*_123_/12πγ_1_(cos θ + 1)]^1/2^. A simple linear regression yields *A*_123_ = 2.79 × 10^–19^ J and *R*^2^ = 0.899; this is shown as the blue dotted line in Figure 3e. Although this relation describes *d*_0_ versus θ fairly well, it has the disadvantage of assuming that all liquid–oxide systems are associated with the same Hamaker constant. An improved approach would be to utilize the relation between the Hamaker constants and the high-frequency dielectric constants in eq 9, which then yields eq 10
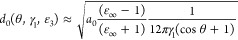
10This approximated expression generates actually very accurate auxiliary gaps as a function of the contact angle (*R*^2^ = 0.997); see red filled circles in Figure 3e. It is applicable for our measured contact angles (blue marks) as well as for the average angles (the two smaller blue marks). If one assumes an error in the calculated dielectric constants of 5% and an error in the measured contact angle of 5°, then the error in the calculated auxiliary gap is 10% for MgO, whereof 8% is from the measured angle. The model involves, however, the constant *a*_0_, which depends on the specific liquid, in our case, molten iron. It would be of interest to know how much the value of *a*_0_ deviates for different liquid–oxide systems.

In addition to the work on the Fe(liq)/oxide systems, we present a brief analysis of the wettability of a liquid tin–bismuth alloy on the oxides. This nontoxic alloy exhibits the advantage of becoming liquid at relatively low temperature and can therefore be subject for experimental investigations of the fluid flow with direct measurement techniques. With this complementary study, it is possible to judge the accuracy of our applied methodology for two rather different types of liquid metals.

We use a similar approach to model the atomic configuration of liquid Sn–Bi as for liquid Fe, however, now using a face-centered cubic Sn_3_Bi structure that mimics an alloy with ∼37 wt % Bi. The wettability of the Sn_3_Bi(liq)/oxides is modeled at *T* = 473 K (200 °C),^61^ at which the liquid surface tension is γ_1_ = 0.46 J/m^2^.^62,63^ For SiO_2_ and ZrO_2_, we considered the trigonal and monoclinic phases, respectively. Due to the strong hygroscopic properties of CaO, the results for Sn_3_Bi(liq)/CaO are not reliable for the present measurement conditions, and we therefore exclude that system. For the four other refractory oxides, the resulting Hamaker constants, contact angles, auxiliary gaps, and works of adhesion are presented in Table 4. One notices that the parameters are quite comparable to the corresponding values for Fe(liq)/oxides; however, *A*_123_ are smaller and *d*_0_ are typically larger. As a consequence, *W* is smaller. This is expected as the surface tension of Sn_3_Bi(liq) is about four times smaller than that of Fe(liq). Therefore, the wettability in terms of the contact angle should be relatively high, and that is also what the sessile drop measurements manifest.

**Table 4:** Similar to Table 3, But Here for Sn_3_Bi(liq)-on-Oxide and at *T* = 473 K

oxide	*A*_123_ (10^–19^ J)	θ (deg)	*d*_0_ (nm)	*W* (J/m^2^)
Al_2_O_3_	2.38	110 ± 2	0.144	0.31
MgO	2.24	86 ± 3	0.109	0.50
SiO_2_(tri)	1.87	80 ± 1	0.096	0.54
ZrO_2_(mono)	2.86	101 ± 3	0.142	0.38

**Figure 4:**
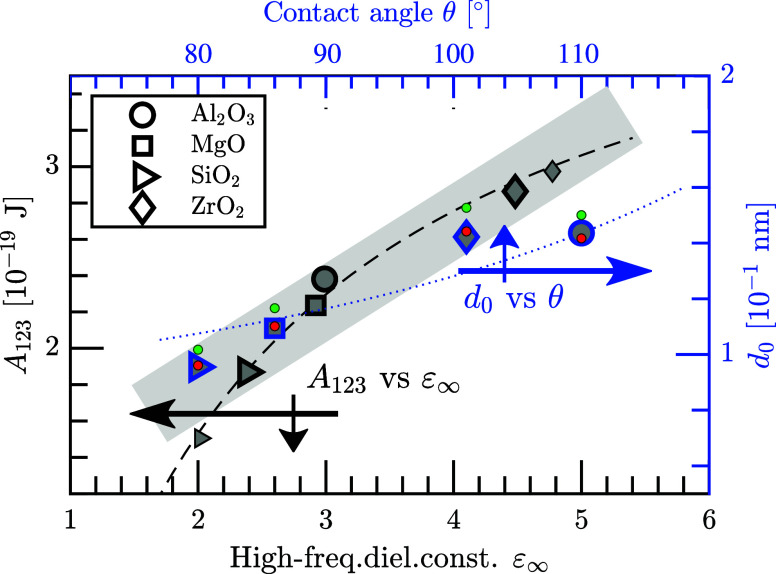
Corresponding graphs to Figure 3b,e, but for the Sn_3_Bi(liq)//oxides and at *T* = 473 K. Black small marks are the high-temperature phases SiO_2_(cub) and ZrO_2_(tetra). Red and green filled circles represent eq 10 with *a*_0_ = 4.59 and 5.15 × 10^–19^ J, respectively.

Likewise the Fe(liq)/oxide systems, the Hamaker constants as a function of the high-frequency dielectric constant are parametrized according to eq 9. The result for Sn_3_Bi(liq)/oxides is *a*_0_ = 4.59 × 10^–19^ J with *R*^2^ = 0.978, which generates the black dashed line in Figure 4. The corresponding behavior of *d*_0_ versus θ, described by eq 10 (red filled circles in the figure), is as good as for the Fe(liq)/oxides (*R*^2^ = 0.995), whereas using a constant value of the Hamaker constant fails to a large extent (blue dotted line; *R*^2^ = 0.817). Actually, the relation in eq 10 (based on eq 9) holds with reasonable accuracy for all considered liquid–oxide systems with one and the same constant. To exemplify the statement, the green filled circles in Figure 4 are the results when using *a*_0_ = 5.15 × 10^–19^ J that was obtained for the Fe(liq)/oxide systems. Further, a regression analysis that includes data from both liquids yields *a*_0_ = 5.0 × 10^–19^ J with *R*^2^ = 0.926. That fit could serve as a crude approximation to be valid for the two molten metals considered here, all five refractory oxides, and for angles ranging at least 80° ≲ θ ≲ 140°. The temperature dependence (see eq 7) has only a moderate impact, as it is compensated by the step lengths of the Matsubara frequencies. However, we want to emphasize that an accurate and more secure determination of the cross-gap interactions requires proper and consistent modeling of Casimir–Lifshitz free energies.

## Conclusions

In this study, we explore the wettability of liquid iron on the five most common refractory oxides, namely, Al_2_O_3_, CaO, MgO, SiO_2_, and ZrO_2_, utilizing a sessile drop technique and first-principles modeling of the cross-interface interactions. The crystal structures of these oxides are analyzed through room-temperature X-ray, verifying the relaxed structures from DFT calculations. Thereafter, the complex dielectric functions of these compounds are computed, and the dielectric constants for both the room-temperature phases and the high-temperature phases are presented. Moreover, we model the dielectric response function of liquid Fe at *T* = 1823 K, whereupon confirming that the spectrum agrees with available experimental data. This model of Fe(liq) significantly simplifies the theoretical exploration of the liquid’s behavior under high-temperature conditions. By combining these atomistic DFT calculations with the macroscopic Casimir–Lifshitz forces, we calculated cross-interface interactions for the Fe(liq)/oxide systems at 1823 K, introducing the auxiliary gap *d*_0_ of the interlayer to link the work of adhesion *W* to the Hamaker constant *A*_123_. The macroscopic three-layer system, denoted Fe(liq)//oxide, is foundational for describing also particle–particle and particle–nozzle interactions in order to simulate agglomeration and nozzle clogging in steel casting processes. It is generic in the sense that it can be applied to other macroscopic liquid–solid systems. Complementing the macroscopic view, the DFT calculations come into play, aiming at a microscopic description of these interactions. These calculations provide thereby an in-depth perspective of the intermolecular interactions between Fe(liq) and the refractory oxides. The resulting estimate of the Hamaker constants for the material systems is a pivotal step in comprehending their interfacial interactions. We explain that *A*_123_ depends predominantly on the high-frequency dielectric constant, ε_∞_, although a full description of the dielectric functions is required for an accurate calculation. Employing only the static dielectric constants in a simplified model of the Hamaker constant would result in incorrect conclusions. With the Casimir–Lifshitz forces, and specifically the Hamaker constants, the wettability of the Fe(liq)/oxide systems is explored by means of Young’s model. The equation bridges the contact angle, an indicative measure of wettability, with the interfacial energies of solid, liquid, and vapor phases. The contact angle data are measured with a sessile drop method at 1823 K, which provides an empirical complement and support to the theoretical models.

**Figure 5:**
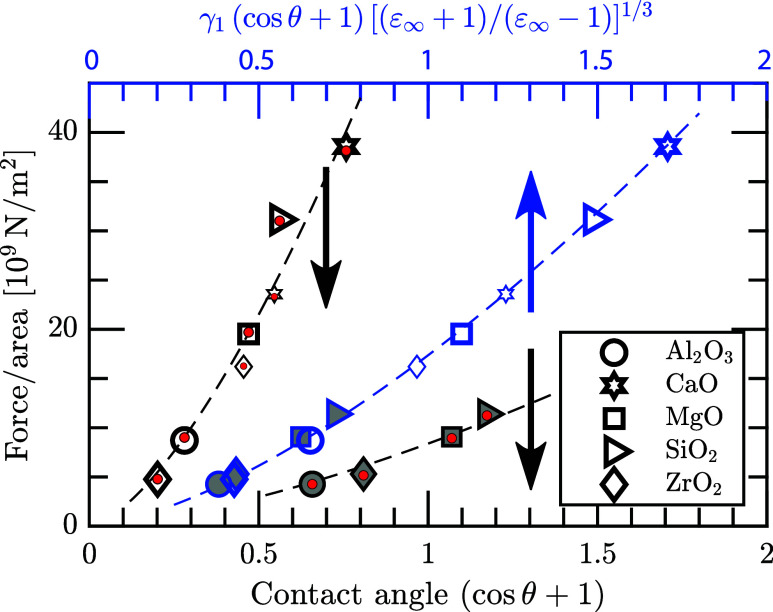
Forces per unit area for Fe(liq)//oxides (open marks) and Sn_3_Bi(liq)//oxides (gray filled marks). Black dashed lines indicate that the forces go roughly as (cos θ + 1)^3/2^. Red circles include eq 10 with *a*_0_ = 5.15 and 4.59 × 10^–19^ J for Fe(liq) and Sn_3_Bi(liq), respectively. Blue dashed line is the best fit to eq 9 for both liquids, i.e., *a*_0_ = 5.0 × 10^–19^ J and by incorporating also the liquids’ surface tension in the model: γ_1_ = 1.843 and 0.460 J/m^2^, respectively.

A key finding of our research is the variance in the value of the spatial auxiliary gap, *d*_0_, for the five systems at *T* = 1823 K, indicating differences in the cross-interface interactions. We emphasize that a consistent model of the auxiliary gaps is needed to accompany the Hamaker constants. The reason is that the relevant physical property for the considered material system is actually the work of adhesion, which involves both the Hamaker constant and the gap, i.e., *W* ∝ *A*_123_/*d*_0_^2^. Utilizing the Hamaker constants in models of particle-on-nozzle stiction or particle–particle agglomeration therefore also requires a proper determination of *d*_0_. By comparing the results for the Fe(liq)/oxide systems with the corresponding results for the Sn_3_Bi(liq)/oxides, we demonstrate that this auxiliary gap depends not only on the oxide but also on the type of liquid. Hence, a crude model of *d*_0_, for instance, a constant value independent of the oxide, could yield inaccurate conclusions. However, for the two liquids and the five oxides, there is a common characteristic. In Figure 5, the forces are depicted for the Fe(liq)/oxides as well as for the Sn_3_Bi(liq)/oxides. From *F*_0_ = 2*W*/*d*_0_ = 2γ_1_(cos θ + 1)/*d*_0_ and eq 8, the forces should behave approximately as (cos θ + 1)^3/2^. This is also what is obtained; see the two black dashed lines for Fe(liq) and Sn_3_Bi(liq) in the figure. Furthermore, if one incorporates eq 10 and the liquids’ surface tension in the model, the cross-interface forces can approximately be described as
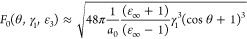
11The characteristic of eq 11 with *a*_0_ = 5.0 × 10^–19^ J and *R*^2^ = 0.999 is represented for both liquids by the blue dashed line and upper axis in Figure 5.

One reason that eq 11 with a constant *a*_0_ is valid for all considered liquid//oxide layers in this study is that eq 9 can be applied for both liquids through ε_1_ ≫ ε_2_ and ε_3_ ≳ ε_2_. This is a reasonably good approximation for the dielectric functions up to ∼10 eV; see Figure 2b. Thereby, the value of the Hamaker constant does not depend on other properties of the liquid metals, apart from the very large ε_1_ due to the Drude contribution for metals. However, to describe the chemical physics of the interfacial system, the auxiliary gap also has to be included. For example, the cross-interface forces in eq 11 depend on *d*_0_, and this can be accounted for by the surface tension of the liquid, which in turn depends implicitly on the liquid’s dielectric response. Thus, the simplified model of eq 9, omitting the liquid’s property, is not enough to analyze wettability, agglomeration, etc. Another reason that eq 9 is valid with reasonable accuracy for all systems is that the oxides have comparable band gap energies. Thereby, the gradual decline of the dielectric responses for energies above ∼6 eV is similar for all oxides. To exemplify this relevance, we construct a hypothetical dielectric function, which is similar to that of a true oxide, but associated with a smaller band gap energy. More concretely, we calculated the dielectric function of Al_2_O_3_, employing the PBEsol functional but excluding the band gap correction. The gap energy is then underestimated by 2.7 eV. Smaller gap yields larger ε_∞_, and therefore the dielectric response is scaled by a constant so that ε_∞_ agrees with the value for true alumina. (For simplicity in this example, we neglect to correct the sum rule.) As a result, the calculated Hamaker constant of the hypothetical oxide is 2.53 × 10^–19^ J, which is 8% smaller than the value for true alumina. The error is perhaps not devastating for theoretically analyzing the wettability, considering the accuracy of corresponding measurements, but it illustrates that one should handle the approximated relation in eq 9 with care.

The insights gained in this work are particularly relevant for processes involving liquid Fe and refractory materials where the wettability plays a significant role, specifically for optimizing steel casting and ceramic manufacturing. The synergy between the theoretical modeling and the experimental validation, as exemplified in this study, underscores a robust approach for future studies in the field. Merging the DFT calculations into the modeling of the Casimir–Lifshitz dispersion forces is demonstrated to provide an accurate as well as accessible methodology in determining the interaction parameters, offering an understanding of the complex relationships between molecular and macroscopic properties. We advocate for further and extended research into the temperature-dependent properties of other material systems, which would enrich the understanding of material behaviors under extreme conditions.

## Supporting Information Available

Experimental setup of the furnace used for the contact angle measurements, comparison of presently and previously reported contact angles, experimental and theoretical X-ray diffraction patterns, and crystal structures considered for DFT calculations (PDF)
