# Correction to “Exploring Wettability of Liquid Iron on Refractory Oxides with the Sessile Drop Technique and Density Functional-Derived Hamaker Constants”

**DOI:** 10.1021/acsami.6c02387

**Published:** 2026-02-13

**Authors:** Sudhanshu Kuthe, Mathias Boström, Wen Chen, Björn Glaser, Clas Persson

In the original published paper, a correction is needed to M. Boström’s acknowledgments:

M.B.’s contributions to this research are part of Project No. 2022/47/P/ST3/01236 cofunded by the National Science Centre and the European Union’s Horizon 2020 Research and Innovation Program under the Marie Sklodowska-Curie Grant Agreement No. 945339. Institutional and infrastructural support for the ENSEMBLE3 Centre of Excellence was provided through the ENSEMBLE3 project (MAB/2020/14) delivered within the Foundation for Polish Science International Research Agenda Programme and cofinanced by the European Regional Development Fund and the Horizon 2020 Teaming for Excellence initiative (Grant Agreement No. 857543), as well as the Ministry of Education and Science initiative “Support for Centres of Excellence in Poland under Horizon 2020” (MEiN/2023/DIR/3797).

