# Supplementary material for: Exploring Wettability of Liquid Iron on Refractory Oxides with the Sessile Drop Technique and Density Functional-Derived Hamaker Constants
Source: ACS Appl Mater Interfaces. 2025 Feb 28;17(10):16173–86. doi: 10.1021/acsami.4c21877 (PMC11913070; doi:10.1021/acsami.4c21877)
Supplement: Supplementary file 1 — am4c21877_si_001.pdf [file am4c21877_si_001.pdf]

SUPPORTING INFORMATION
Exploring Wettability of Liquid Iron on Refractory Oxides with the Sessile Drop Technique and Density Functional-Derived Hamaker Constants

Sudhanshu Kuthe,*,† Mathias Boström,‡,§ Wen Chen,¶,‖ Björn Glaser,† and Clas Persson*,†

†*Department of Materials Science and Engineering, KTH Royal Institute of Technology, SE-100 44 Stockholm, Sweden*

‡*Centre of Excellence ENSEMBLE3 Sp. z o. o., Wolczynska Str. 133, 01-919, Warsaw, Poland*

¶*UniversalLab GmbH, Park Innovaare: deliveryLAB, 5234 Villigen, Switzerland*

§*Chemical and Biological Systems Simulation Lab, Centre of New Technologies, University of Warsaw, Banacha 2C, 02-097 Warsaw, Poland*

‖*Yangtze Delta Region Institute of Tsinghua University, Zhejiang, 314006, China*

E-mail: kuthe@kth.se; claspe@kth.se

Experimental work

Sessile drop experiments

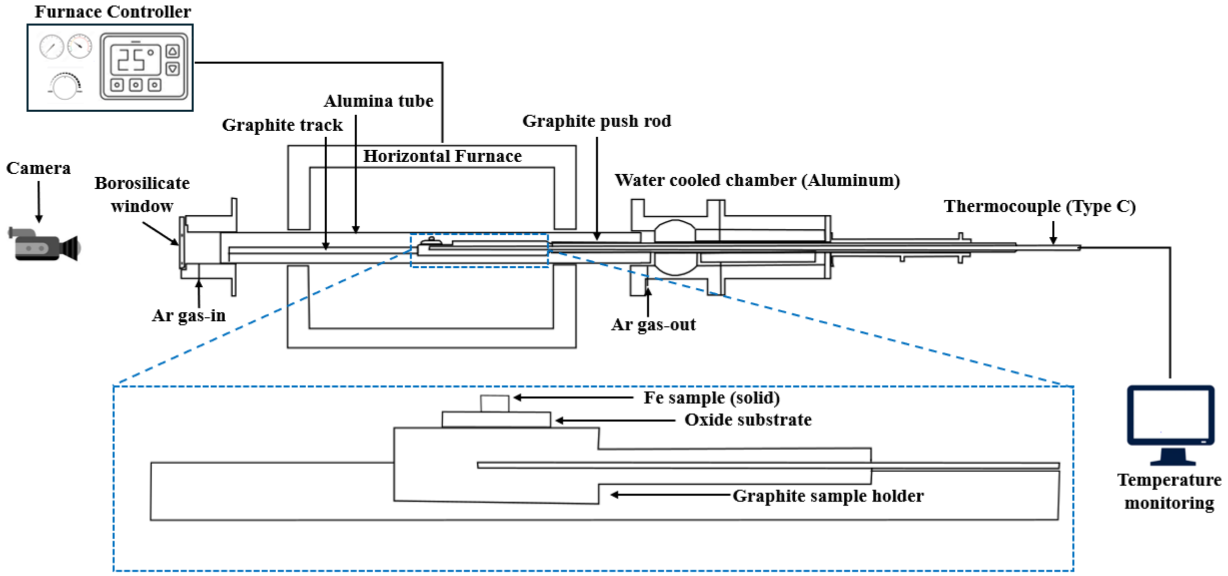

Figure S1: Experimental setup of the furnace used for contact angle measurement,

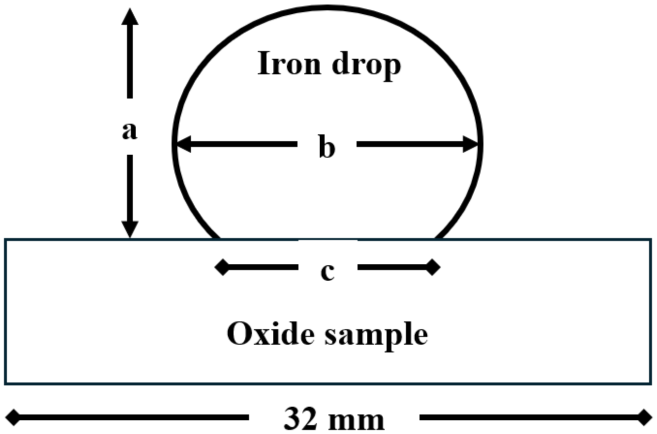

System	a	b	c
Fe-Al_2O_3	6.17	8.66	6.33
Fe-MgO	6.20	10.28	8.86
Fe-SiO_2	5.71	8.22	8.30
Fe-CaO	6.63	7.56	7.56
Fe-ZrO_2	8.60	8.39	6.03

Figure S2: Three parameters describing the size of the Fe(liq) droplet: **a** represents the height, and **b** is the maximum diameter of the drop, and **c** measures the contact length where the drop meets the oxide substrate; all parameters are specified in millimeters.

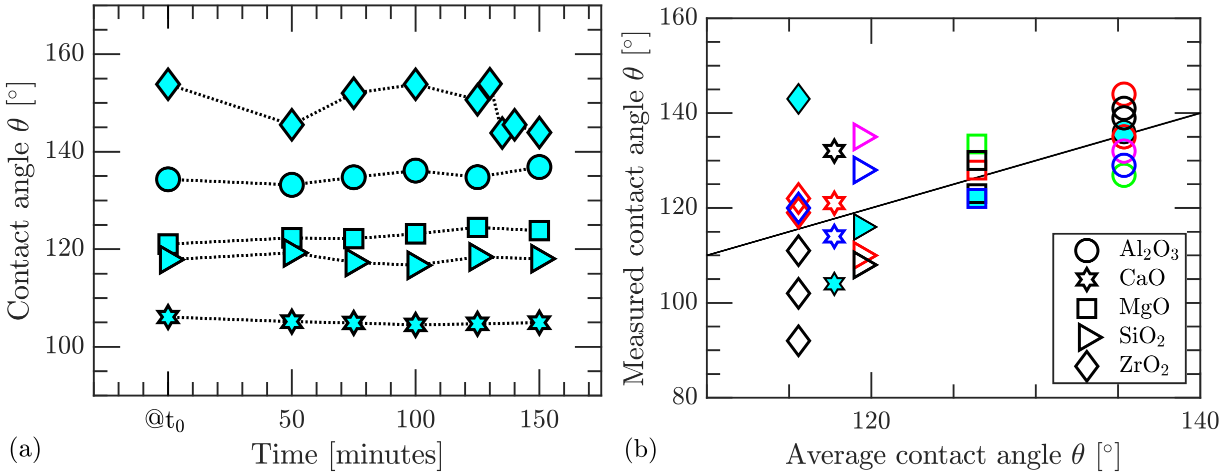

Figure S3: (a) Measured contact angles over time using the low-bond axisymmetric drop shape analysis. (b) Deviation of the measured contact angles from the average value. Black line indicates a perfect match between them. Turquoise filled marks represent present results. Data from the literature are from Nakashima et al. (red open marks; a collection of data), Shen et al. (green), Kapilashrami, et al. (magenta), Samsonov (black; a collection of data), and Ogino et al. (blue); see main text for references. Shen et al. report a deviation of less than 1.5° for liquid iron with 0.0018% Al on MgO and Al_2O_3, measured over ~ 100 minutes at 1873 K. The data from Ogino et al. and for SiO_2 from Kapilashrami et al. are the results at the initial time. Kapilashrami et al. expect that their observed rapid decline in contact angle is due to formation of Fe_2SiO_4. They indicate error bars of about $\pm 3°$ to $\pm 5°$.

X-ray diffraction analysis

X-ray diffraction (XRD) analysis was specifically conducted on refractory oxide powder particles. The primary purpose of XRD was to validate the lattice constants obtained from the DFT calculations. The XRD machine used for the analysis was bruker d2 phaser.

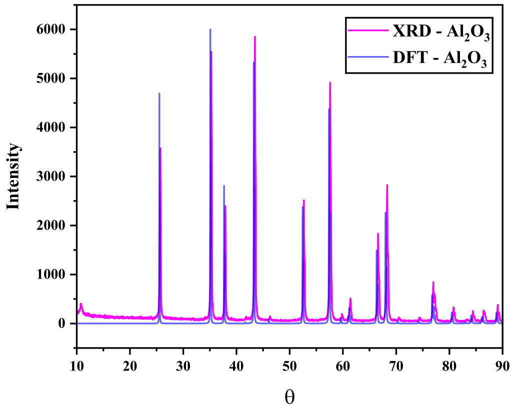

Figure S4: Powder diffraction pattern for Al_2O_3 from XRD measurement and DFT.

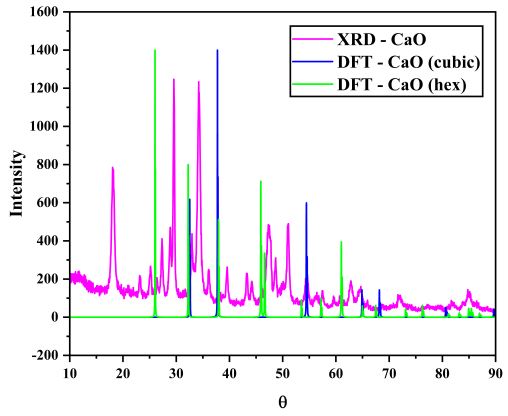

Figure S5: Powder diffraction pattern for CaO from XRD measurements and DFT.

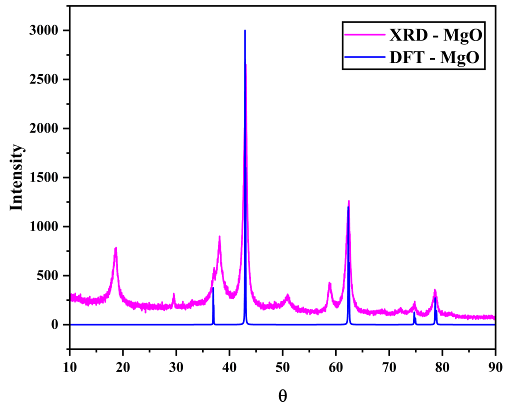

Figure S6: Powder diffraction pattern for MgO from XRD measurement and DFT.

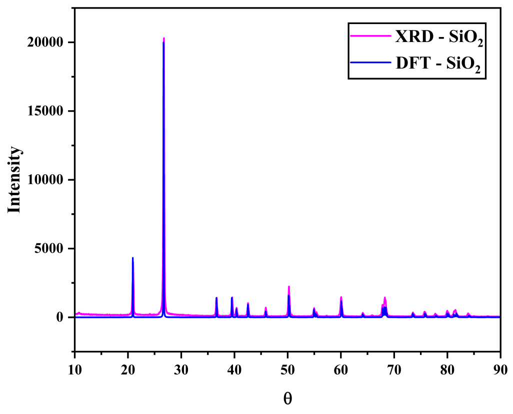

Figure S7: Powder diffraction pattern for SiO_2 from XRD measurement and DFT.

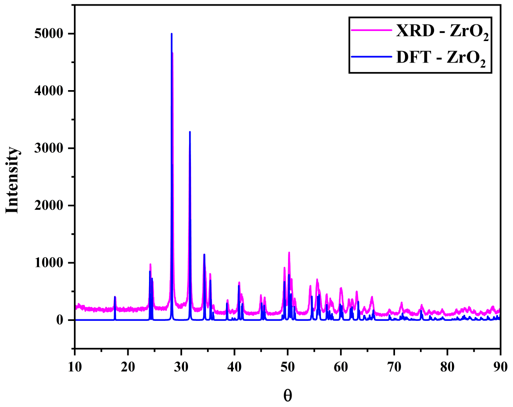

Figure S8: Powder diffraction pattern for ZrO_2 from XRD measurement and DFT.

Structures considered for the DFT calculations

Figures have been generated with the software package Visualization for Electronic and STtructural Analysis; K. Momma and F. Izumi, "VESTA 3 for three-dimensional visualization of crystal, volumetric and morphology data," J. Appl. Crystallogr., 44, 1272–1276 (2011).

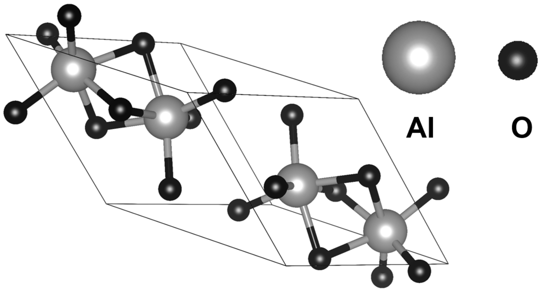

Figure S9: Crystal structure of Al_2O_3 considered for the DFT calculations.

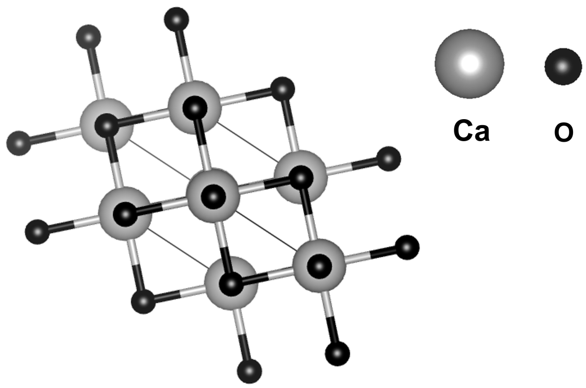

Figure S10: Crystal structure of CaO considered for the DFT calculations.

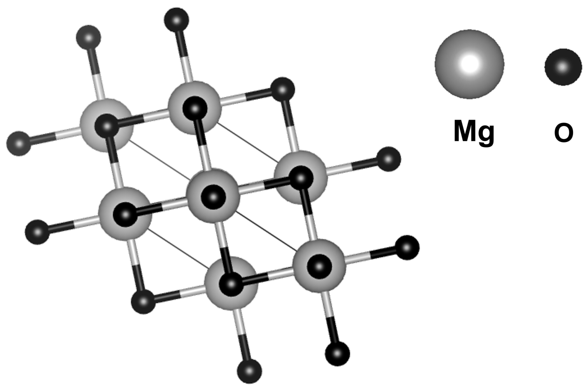

Figure S11: Crystal structure of MgO considered for the DFT calculations.

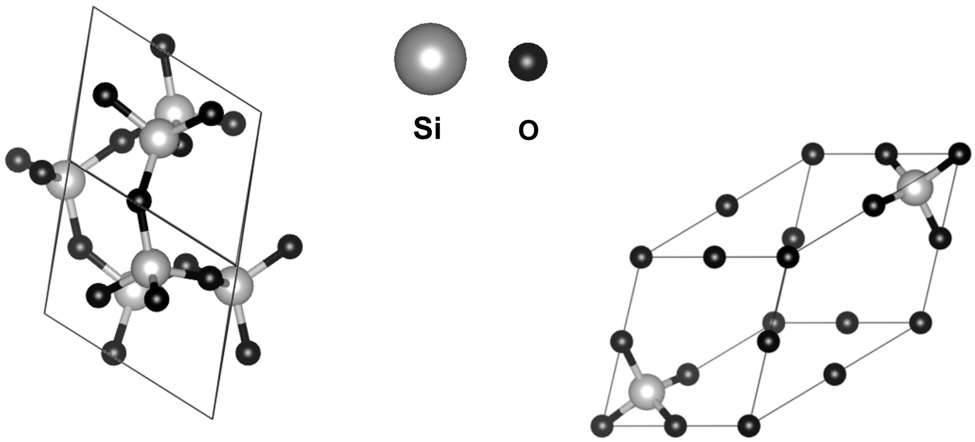

Figure S12: Crystal structures of room-temperature (left) and high-temperature (right) phases of SiO_2 considered for the DFT calculations.

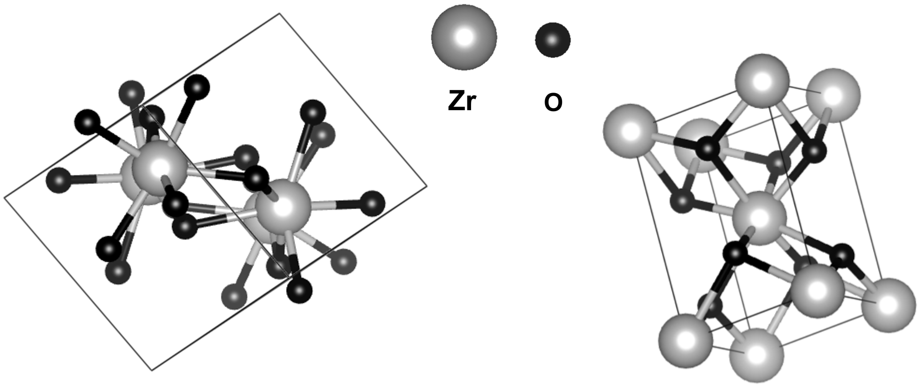

Figure S13: Crystal structure of room-temperature (left) and high-temperature (right) phases of ZrO_2 considered for the DFT calculations.
